# COVID-19: FAQs—Congenital Heart Surgery Recovery and Defining a “New Normal”

**DOI:** 10.1177/2150135120934741

**Published:** 2020-07-14

**Authors:** Joseph A. Dearani, Elizabeth H. Stephens, Kristine J. Guleserian, David M. Overman, Carl L. Backer, Jennifer C. Romano, James D. St Louis, George E. Sarris, Emile Bacha, James S. Tweddell

**Affiliations:** 1 Department of Cardiovascular Surgery, 4352 Mayo Clinic, Rochester, MN, USA; 2 Department of Congenital Heart Surgery, Medical City Children’s Hospital, Dallas, TX, USA; 3 Division of Cardiac Surgery, Children’s Minnesota, Minneapolis, MN, USA; 4 Section of Pediatric Cardiothoracic Surgery, Kentucky Children’s Hospital, Lexington, KY, USA; 5 Department of Cardiac Surgery, 21634 C. S. Mott Children’s Hospital, University of Michigan, Ann Arbor, MI, USA; 6 Department of Pediatric Cardiac Surgery, University of Missouri–Kansas City School of Medicine, Kansas City, MO, USA; 7 Department of Pediatric Heart Surgery, Athens Heart Surgery Institute, Athens, Greece; 8 Department of Surgery, Division of Cardiothoracic Surgery, 5798 Columbia University Irving Medical Center/New York–Presbyterian, NY, USA; 9 Department of Surgery, Division of Cardiothoracic Surgery, Cincinnati Children’s Hospital Medical Center and the University of Cincinnati, OH, USA

**Keywords:** COVID-19, congenital cardiac surgery, crisis management

## Abstract

As recovery of congenital heart surgery programs begins during this COVID-19 pandemic, we review key considerations such as screening, protection of patients and health care workers (HCWs), case prioritization, barriers to reactivation, redesign of patient care teams, contribution of telemedicine, modification of trainees’ experiences, preparation for potential resurgence, and strategies to maintain HCW wellness. COVID-19 has tested the resolve and grit of our specialty and we have an opportunity to emerge more refined.

## Introduction

The COVID-19 pandemic has radically changed the world as we once knew it, dramatically impacting our world’s health, finances, and social interactions. In the surgical world, operating rooms have come full circle…from being shut down or reallocated as intensive care units (ICUs) during the peak of the COVID crisis, to periodic use during “curve flattening,” to ramping up as we progress on a path toward a new normal. Terror is fizzling and the crisis feels less forceful. Awe-inspiring individual and team efforts with extraordinary motivation and determination have carried us through, but fear and uncertainty are still present.

It is clear that there is a regional element to the COVID-19 pandemic with some parts of the world, and some regions of large nations such as the United States were substantially more affected than others. As a result, the cardiothoracic surgery recovery from COVID-19 will have different strategies and guidelines in light of these regional variations. It’s time to pause and come to terms with the fact that our previously experienced “normal” is gone, that the future brings a new “normal,” and the next “reactivation” phase of cardiothoracic surgery is an opportunity to improve upon our ability to care for our patients, to examine, adjust, and recalibrate to a new baseline—one that we hope may ultimately be superior to the remarkable reference point that will be left behind.^1^

Wood DA, Mahmud E, Thourani VH, et al. Safe reintroduction of cardiovascular services during the COVID-19 pandemic: guidance from North American Society Leadership. *J Am Coll Cardiol*. 2020.

1. ***What are the considerations and barriers to the recovery phase of surgical services?***

The reintroduction of cardiovascular services must aim to maximize benefits for the population at large, prioritizing procedures that will have the most impact in terms of lives saved, and health-related quality of life preserved or improved, over procedures that will have less impact and/or will benefit fewer people. A systematic approach should be applied that weighs the risk of further treatment delay against the risk of worsening COVID-19 spread. Sufficient resources, including staff, testing, equipment, and personal protective equipment (PPE), must be considered and may remain a barrier for some institutions. Finally, the reintroduction of procedures should be implemented with equity across patient populations regardless of ethnicity or ability to pay. With these factors in mind, two barriers that deserve emphasis include *trust* of the patient community in the medical profession and *healthcare equity*. The regional aspect of COVID-19 will result in strategies and time lines that differ from city to city, state to state, and country to country, depending on the number of patients affected, resource availability, and recommendations by hospital leadership and government officials. This variability in strategies and time lines, while thoughtfully and appropriately planned, may be perceived by the patient community as chaos and confusion within the medical community. This may result in a lack of trust and confidence in the medical profession and could lead to a desire to avoid surgery (or other invasive procedures) for fear of becoming infected while being an inpatient. The importance of clear, informative communication between the physician and patient/family to instill confidence and convey a sense of security and safety in the hospital environment cannot be overemphasized. Second, health care equity can be challenging. Reactivation strategies should be consistent across ethnicities and socioeconomic classes independent of health care coverage.^1-5^

Wood DA, Mahmud E, Thourani VH, et al. Safe reintroduction of cardiovascular services during the COVID-19 pandemic: guidance from North American Society Leadership. *J Am Coll Cardiol.* 2020.

Chiriboga D, Garay J, Buss P, Madrigal RS, Rispel LC. Health inequity during the COVID-19 pandemic: a cry for ethical global leadership. *Lancet.* 2020. Published online May 15, 2020.

Gawande AA. Amid the coronavirus crisis, a regimen for reentry. *The New Yorker Magazine*. Condé Nast; 2020.

Persad G, Wertheimer A, Emanuel EJ. Principles for allocation of scarce medical interventions. *Lancet.* 2009;373(9661):423-431.

White DB, Lo B. A framework for rationing ventilators and critical care beds during the COVID-19 pandemic. *JAMA.* 2020.

2. ***Protection of patients and health care workers (and other hospital employees with potential patient contact; ie, security, transport, housekeeping, physical plant maintenance and engineering, etc) needs to be addressed during the reactivation of surgical procedures and tests. What are the best approaches to*** ***(1) physical distancing in the hospital, that is, protocols for minimizing nonessential contact between patients and health care workers, and visitor policy, (2) screening strategy for patients and health care workers, and (3) personal protective equipment?***

During the reactivation phase, protection of patients and HCWs must be prioritized and systematized. A screening and retesting plan for all patients and HCWs must be in place and continuously reexamined and modified as new data and testing resources become available. A “health check” for HCWs at hospital entrance (temperature, symptoms) and visitor restrictions should be in place. Specific protocols for visitors remain particularly difficult for the pediatric population who almost exclusively depend on a parent or guardian for psychosocial support during hospitalization. While guidelines are institution-specific and will undoubtedly continue to change, consideration must be made for each patient regarding adequate psychosocial support while minimizing exposure. At this time, most institutions allow one visitor per patient; in some cases, this is one visitor at a time with the ability to alternate between two designated guardians. COVID screening for that visitor depends on resource availability and symptoms, and the management of COVID-positive parents is handled on a case-specific basis. Remodeling patient “pathways” and waiting rooms should be ongoing. Strategies to minimize contact between patients and HCWs utilizing telemedicine and the cumulative number of face-to-face contacts in the perioperative phase should be employed and consolidation of multiple tests/procedures during the shortest time interval should be the goal. However, this must be done in a manner that minimizes depersonalization and avoids imparting fear in patients and families. The choice of polymerase chain reaction (PCR) nasal swabs and saliva or rapid serum antibody tests may vary from institution to institution and should be guided by hospital infectious disease experts and coordinated with regional health officials. At present, PCR is typically required the day before or the morning of surgery. Appropriate PPE is essential to protect HCWs even with asymptomatic patients. This may range from N95 masks routinely for operating room team members to limiting their use to anesthesia team members only, unless COVID positivity is established. The need to protect staff must be balanced against the need to conserve PPE supplies in the event of resurgence. While rules and regulations are certainly key to continuing the practices that have helped curtail the COVID-19 pandemic, fostering a culture in which such practices, which may feel burdensome at times, are embraced cannot be underestimated. As Gawande writes, “People tend to focus on two desires: safety and freedom; keep me safe and leave me alone.” But in these times, we need our community to worry about the safety of others.^1,3,6^

Wood DA, Mahmud E, Thourani VH, et al. Safe reintroduction of cardiovascular services during the COVID-19 pandemic: guidance from North American Society Leadership. *J Am Coll Cardiol.* 2020.

Gawande AA. Amid the coronavirus crisis, a regimen for reentry. *The New Yorker Magazine*. Condé Nast; 2020.

American Medical Association. COVID-19: a physician practice guide to reopening. American Medical Association. Published 2020. Updated May 21, 2020. Accessed May 21, 2020, 2020. https://www.ama-assn.org/delivering-care/public-health/covid-19-physician-practice-guide-reopening

3. ***What are the strategies for selection and prioritization of surgical cases?***

Congenital heart surgery programs are accustomed to the necessity of case selection and prioritization within the confines of a given program’s resources, whether that be for an urgent or emergent case that bumps an elective case or selecting cases based on limited bed availability. While children have been relatively spared from COVID’s clinical impact, and the disease has, so far, had a limited effect on the course of children with congenital heart disease,^7^ case prioritization has drastically changed for congenital heart programs during COVID because of a severe restriction of resources. These restrictions are due to two causes: resources redirected to the care of adult patients with COVID and staffing strategies to decrease exposure risk and COVID spread. In the setting of such limited resources, proper prioritization of surgical cases is essential. Cardiac surgery has inherent immutable risks and substantial potential benefits. The indications for surgery are symptomatic improvement and prevention of premature death. By its very nature, there are no truly elective cardiac surgeries. While it may be suitable to delay surgery in some cases, eventually these operations will become necessary and further delay could risk a patient’s quality of life and survival.

A recent article published jointly in several cardiothoracic surgery journals and promoted on the websites of the European Congenital Heart Surgeons Association (ECHSA), World Society for Pediatric and Congenital Heart Surgery (WSPCHS), and Congenital Heart Surgeons' Society (CHSS) identified multiple factors that must be taken into account in order to prioritize cases including: (1) resource utilization, such as anticipated ventilator duration, ICU stay, blood product usage, and other supplies that are or may become limited, (2) clinical status of the patient and risk of delaying surgery, (3) risk of exposure for the patient, family, and health care staff, and (4) comorbidities and complexity of the procedure with implications on the usage of hospital resources, (5) in teaching hospitals, training may have to be curtailed and the most experienced surgeons used liberally, and (6) the safety of the patient’s social and clinical situation if surgery is delayed. A table from that publication is included here (Table 1) and may help guide us as we begin the recovery phase of pediatric heart surgery.^8^

For patients who require surgery and test positive for COVID and do not yet display antibodies, the decision-making around timing of surgery becomes even more difficult. While ideally surgery should wait until serology testing demonstrates antibodies, or at least until symptoms if present resolve, this is not always possible. Furthermore, it remains unclear to what degree antibodies provide immunity. Each such patient must be considered individually, considering the risks of waiting and the current, albeit continuously changing, knowledge regarding the risks of proceeding with surgery based on the COVID testing results and presence of symptoms.

Expanding to pre-COVID-19 pandemic levels of congenital heart surgery volume might logically be achieved once one or more of the following have occurred: development of a vaccine; effective treatment; reliable, rapid, and widely available testing of both staff and patients; and decreased infection rate in the community to a low level. In the meantime, mitigation efforts such as social distancing, physical barrier creation, frequent hand washing, surface cleaning, masking, and preoperative patient testing will need to continue in the setting of reactivation. In terms of case selection, the pool of cases will expand from emergent and urgent, to “high-priority elective” operations. Increasing surgical volume where social distancing is our primary mitigation strategy requires limiting person-to-person exposure and will remain a challenge in the health care environment. Hospitals were designed to maximize occupancy, not to provide social distancing. If we are to provide the same volume of service while honoring social distancing, institutions may need to consider the addition of routine weekend surgery and/or increasing operating room utilization such as adding evening shifts.

As the pandemic evolves, our work strategies will change, with an aim to strike a risk–benefit balance for healthcare providers and patients. Short of achieving those goals that would permit a pre-COVID-19 level of social interaction, we will continue to require altered work schedules that will necessarily limit our ability to care for patients. This will depend on even more careful individual assessment of patient risk and prioritization (Table 1).

**Table 1.** Congenital Heart Lesion and Surgical Prioritization during COVID-19.^a^

Patient	**Emergent** (24-48 Hours of Diagnosis When Adequate Resources)	**Urgent** (Within 1-2 Weeks When Adequate Resources)	**High Priority Elective** (>2 Weeks When Adequate Resources)
**Neonate**	*Note: Timing for categories will depend on resources available, institutional protocols, and other pending cases*		
Shunts: right → left			
TAPVC/cor triatriatum	Obstructed	Increasing gradient	
TGA		<1 week if IVS	2-4 weeks if VSD
Truncus arteriosus			If stable
Tetralogy of Fallot	Spelling/deep cyanosis	Symptomatic	
Regurgitant lesions			
Ebstein anomaly		Refractory medical management	
Obstructive lesions			
Coarctation	Shock unable to stabilize on PGE	If able to stabilize on PGE	
Critical aortic stenosis	Shock unable to stabilize on PGE	If able to stabilize on PGE	
PGE-dependent pulmonary blood flow			
PA/IVS		If PDA stent not available	
PGE-dependent systemic blood flow			
HLHS	Intact, restrictive atrial septum if BAS not available	Case and surgeon dependent	Case and surgeon dependent
Other			
Shunt	Shunt thrombosis	Shunt stenosis	
Arrhythmias	Symptomatic congenital heart block unable to medically manage/externally pace		
ALCAPA	Once medically stabilized		
**Infant**			
Shunts: left → right			
VSD		Symptomatic CHF on medical management	Failure to thrive
Shunts: right → left			
Tetralogy of Fallot		Symptomatic (spells, cyanosis) on medical management	
Regurgitant lesions			
AVSD			Tri21 with pulmonary overcirculation, consider age of patient to optimize repair, significant regurgitation unable to manage medically
Ebstein anomaly			Increasing right-sided heart failure on medical management
Mitral regurgitation			Symptomatic CHF on medical management
Aortic regurgitation	Acute, hemodynamically unstable		Enlarging LV, decreasing LVEF, symptoms
Obstructive lesions			
Valve prosthesis	Thrombosed prosthesis		
AS/LVOTO			Decreasing LVEF, symptoms
RVOTO			Decreased RV function
Other			
Shunt	Shunt thrombosis	Shunt stenosis	
DCM/HF		CHF failing medical management	Failure to thrive
BDCPA candidate			Increasing cyanosis with current shunt, shunt stenosis

Abbreviations: ALCAPA, anomalous left coronary artery from the pulmonary artery; AS, aortic stenosis; AVSD, atrioventricular septal defect; BDCPA, bidirectional cavopulmonary anastomosis ; CHF, congestive heart failure; DCM/HF, dilated cardiomyopathy/heart failure; HLHS, hypoplastic left heart syndrome; LV, left ventricle; LVEF, left ventricular ejection fraction; LVOTO, left ventricular outflow tract obstruction; PA/IVS, pulmonary atresia with intact ventricular septum; PGE, prostaglandin E; RV, right ventricle; RVOTO, right ventricular outflow tract obstruction; TAPVC, total anomalous pulmonary venous connection; TGA, transposition of great artery; VSD, ventricular septal defect.

^a^ Reproduced from Stephens et al.^8^

Shekerdemian LS, Mahmood NR, Wolfe KK, et al. Characteristics and outcomes of children with coronavirus disease 2019 (COVID-19) infection admitted to US and Canadian pediatric intensive care units. *JAMA Pediatr.* 2020.

Stephens EH, Dearani JA, Guleserian KJ, et al. COVID-19: crisis management in congenital heart surgery. *World J Pediatr Congenit Heart Surg.* 2020; 11: 395-400.

4. ***What is the role of telemedicine?***

There is no doubt that reliance on telemedicine during reactivation and even post-COVID will be a more integral part of health care, including the care of patients with congenital heart disease. Not only does it decrease exposure risk, which is critical during this COVID pandemic, but it provides convenience to our patients and minimizes geographical limitations, which will remain an important benefit post-COVID. In the setting of decreased resources during COVID, telemedicine has been an important facet of triaging surgical patients and can play an essential role in that manner moving forward. Challenges remain, however, regarding telemedicine, including understanding its limitations and optimizing its role: We must decipher which patients cannot be adequately assessed using telemedicine and would benefit from an in-person visit despite slightly increased risk to the patient and providers. We will need to determine how telemedicine technology and providers’ and patients’ skills using telemedicine can be improved to allow effective communication and an optimized patient experience. We must guard against the implementation of telemedicine reinforcing or propagating health care disparity, whether that be access to resources or digital literacy. Payment structure and credentialing must be examined and altered to support effective telemedicine. All of these challenges will be answered in time since it is clear that many aspects of telemedicine are here to stay.^9,10^

Hollander JE, Carr BG. Virtually perfect? Telemedicine for COVID-19. *N Engl J Med.* 2020;382(18):1679-1681.

Ohannessian R, Duong TA, Odone A. Global telemedicine implementation and integration within health systems to fight the COVID-19 pandemic: a call to action. *JMIR Public Health Surveill.* 2020;6(2):e18810.

5. ***Will the model of team-based care be different?***

At many institutions during the peak of the COVID-19 pandemic, the care model drastically and dynamically changed as staff resources were flexed to provide needed care. Even in institutions not as severely impacted, strategies were employed to decrease exposure and preserve the workforce. During reactivation, the redeployment of staff into other clinical areas will no longer occur, but some key principles still remain relevant, particularly given the risk of resurgence. Many of these alternate models have provided an opportunity to critically reevaluate workflow for efficiency and reduced redundancy. Careful, thoughtful planning regarding the optimal use of staff will continue to be essential, so as to not duplicate work and/or increase unnecessary exposure. Block schedules should be considered to help preserve the workforce. Selective application should be applied for some hospitals to move to a 7-day work week to decrease the population density of various areas of the hospital, for example, parking, offices, lab/diagnostic testing, pharmacy, and so on. Certain elements of patient care will likely remain virtual, such as computer-generated visits to triage patients, and for pre- and postoperative visits. Many of the nonclinical support staff may continue to work remotely for the foreseeable future, some indefinitely. Sustained surveillance for symptoms, appropriate PPE, and testing remain important. While constant emphasis on physical distancing is essential during reactivation, the intense collaboration between medical and surgical specialties that has been required to face the many challenges of the pandemic as a united front thus far will strengthen preexisting strong bonds between all members of the care team moving forward.^1,8^

Stephens EH, Dearani JA, Guleserian KJ, et al. COVID-19: Crisis management in congenital heart surgery. *World J Pediatr Congenit Heart Surg.* 2020; 11: 395-400.

Wood DA, Mahmud E, Thourani VH, et al. Safe reintroduction of cardiovascular services during the COVID-19 pandemic: guidance from North American Society Leadership. *J Am Coll Cardiol.* 2020.

6. ***How should the trainees’ experience be modified during the recovery phase?***

While trainees in all specialties require clinical experience as part of their education, the core component of training in surgery is “hands-on,” involving real patients in the operating room. In other specialties, simulation and other remote learning tools may compensate for diminished in-person clinical experience; this is not the case for those learning congenital cardiac surgery. The question then becomes how to optimize the training experience while minimizing risk to the trainee, staff, and the patient. Additionally, the trainees’ role in the staffing model for the clinical service needs to be considered. Each institution should evaluate their staffing model and the educational needs of each trainee, for example, case log to date to identify a need for more experience in a certain procedure. Deficiencies in case logs should be coordinated with the operations performed in the setting of the pandemic. In contrast, if the trainee has had adequate experience and shown competence in a specific operation, then consideration should be given to whether additional exposure of both the trainee and patient is necessary or important. With regard to service staffing, duplicative efforts by trainees should be avoided, so one resident plays the primary role of inpatient needs, rounding, and so on, and rotation schedules are planned with the objective of decreasing HCW-patient exposure. The role of physician extenders to help cover patient care needs is essential to optimize the trainees’ focus on educational opportunities while ensuring adequate coverage of service needs. Institutions must provide adequate PPE as well as training in its proper use. Reallocation of trainees to other departments should be done carefully so the trainee’s skill set matches the patient needs. Optimization of web-based learning and simulation can supplement but not replace hands-on training in our specialty. Finally, the American Board of Thoracic Surgery and the Accreditation Council for Graduate Medical Education will consider the educational experience and case logs for individual trainees during this period. While some flexibility with regard to case requirements will be considered, major deficiencies will likely result in the need for additional training to optimize and ensure competence for certification.^11-15^

Harrington RA, Elkind MSV, Benjamin IJ. Protecting medical trainees on the COVID-19 frontlines saves us all. *Circulation.* 2020;141(18):e775-e777.

Kogan M, Klein SE, Hannon CP, Nolte MT. Orthopaedic education during the COVID-19 pandemic. *J Am Acad Orthop Surg.* 2020.

Porpiglia F, Checcucci E, Amparore D, et al. Slowdown of urology residents’ learning curve during the COVID-19 emergency. *BJU Int.* 2020.

Fuller S, Vaporciyan AA, Dearani JA, Stulak JM, Romano JC. COVID-19 disruption in cardiothoracic surgical training: an opportunity to enhance education. *Ann Thorac Surg.* 2020. IN PRESS.

Caruana EJ, Patel A, Kendall S, Rathinam S. Impact of Covid-19 on training and wellbeing in subspecialty surgery: a national survey of cardiothoracic trainees in the United Kingdom. *J Thorac Cardiovasc Surg.* 2020. IN PRESS.

7. ***What is the role of our governing organizations during the pandemic, for example, Congenital Heart Surgeons’ Society, Society of Thoracic Surgeons?***

The specialty of congenital heart surgery is very small relative to the remainder of the cardiothoracic field. As such, our professional organizations play an increasingly important role in providing the sense of community necessary among our colleagues for sharing information and ideas and for support. Especially in times of crisis, the connectivity provided by our professional organizations, for example, the CHSS, Society of Thoracic Surgeons, American Association for Thoracic Surgery, European Congenital Heart Surgeons Association, and World Society for Pediatric and Congenital Heart Surgery, is essential. These organizations provide us with ongoing, up-to-date information on the impact of COVID-19 on our specialty, guidance on how best to care for our patients and for each other, and advocacy to make sure our voices are heard. The social distancing mandated by this pandemic, however, has greatly impacted and, in most cases, halted our normal interactions with these organizations through annual meetings and educational opportunities. This has the potential to undermine our sense of community and involvement. It is incumbent on our professional organizations to adapt to the rapid changes our world has experienced, with increased dependence on electronic opportunities for “face time” with our peers. In addition to the dramatic and essential increase in email and online communication, the value of seeing one another and interacting via live video webinars, meetings, and conference calls, now more than ever, is pivotal in maintaining our community network. Undoubtedly, the role of virtual meetings and optimizing their delivery will become integral to our specialty at least in the short-term and potentially play a role longer-term even as restrictions are lifted. When properly orchestrated, these meetings allow for even increased worldwide participation and collaboration, which can only help our specialty. As our organizations have quickly adapted in the ongoing development of a “new normal,” they will need to critically evaluate how we interact with each other and our organizations going forward. It is important for members to understand the constraints being faced by our organizations, as with all impacted by this crisis, including financial challenges, major shifts in staffing models, and ever-changing restrictions in the face of an uncertain future. The corollary is the necessity for our organizations to be a strong presence for the membership with reassurance that despite these challenges, they are working hard to continue to support their mission and membership. Travel restrictions will remain for an indeterminate time, limiting the ability of our profession to meet collectively, which is essential for collaboration, innovation, education, research, and advocacy. Organizations need to determine and rapidly implement strategies to maintain our professional connectivity and assure the membership that, despite restrictions, they remain the stalwart leaders of our profession.

Wood DA, Mahmud E, Thourani VH, et al. Safe reintroduction of cardiovascular services during the COVID-19 pandemic: guidance from North American Society Leadership. *J Am Coll Cardiol.* 2020.

8. ***What institutional resource strategies should be in place in the event of a resurgence?***

A key consideration in the reactivation phase is the significant risk of resurgence. Institutions must be vigilant and commit to constant surveillance for indications of COVID-19 resurgence in the local/regional area and within their institution. The institution should make it clear to staff and the community when reopening activities that there is a possibility of resurgence that would require reinstituting restriction measures. Preparations for this should include strategies to fluidly progress from one stage of reopening to another and then back as need be. Case volume and prioritization may need to fluctuate depending on the local incidence of COVID-19 resurgence. Models for optimizing staff in the operating room and ICU may also need to change depending on local occurrence and resurgence. Screening protocols should evolve based on hospital protocols and government guidance. The shortage of PPE experienced by many institutions during the peak of the pandemic should prompt programs to examine prior data regarding their PPE requirements and to plan sufficient inventory accordingly. Institutions should consider COVID pathways and non-COVID pathways to allow non-COVID patients to receive treatment despite resurgence. The new “normal” is a changing target that requires constant readiness and adaptability.^1^

Wood DA, Mahmud E, Thourani VH, et al. Safe reintroduction of cardiovascular services during the COVID-19 pandemic: guidance from North American Society Leadership. *J Am Coll Cardiol.* 2020.

9. ***Should contingency plans for any resurgence involve your hospital only or include collaboration with other local and regional hospitals, particularly hospitals that may normally be viewed as competitors for your program?***

Beyond programmatic and institutional considerations, it is imperative that congenital heart surgery programs continue to safeguard their ability to fulfill their collective responsibility as a fundamental public health resource. The median number of surgeons at programs in the United States is three, and in many locales, that number is one or two. Beyond the operating room, the ICU and other critical multidisciplinary team members may also have staffing models with limited redundancy. Thus, neighboring programs and programs with regional overlap are encouraged to maintain contingency plans for assisting one another when capacity challenges arise, even if these programs are considered competitors under more normal circumstances. Provision should be made for temporary or emergency privileging, and frequent and transparent conversation regarding emerging personnel or supply shortages is crucial.^8,16^

Stephens EH, Dearani JA, Guleserian KJ, et al. COVID-19: crisis management in congenital heart surgery. *World J Pediatr Congenit Heart Surg.* 2020; 11: 395-400.

Morales DL, Khan MS, Turek JW, et al. Report of the 2015 Society of Thoracic Surgeons Congenital Heart Surgery Practice Survey. *Ann Thorac Surg.* 2017;103(2):622-628.

10. ***What approaches should be implemented to address and maintain physician and health care workers wellness and minimize post-traumatic stress disorder?***

One of the dangers of traumatic and world-changing events like 9/11—*and now the COVID-19 crisis—*is the trigger of sudden, intense feelings of helplessness and hopelessness. Although *physical* safety of physician and HCWs has been a focus and is essential to managing the evolving pandemic, our *psychological*, *emotional*, and *spiritual* well-being are also key to functioning under adverse conditions. These include unprecedented ethical and moral dilemmas that will inevitably result in some degree of burnout and mental health–related problems. The mental health collateral damage from COVID-19 has even been cited as a second expected pandemic.^17^ Maintaining healthy eating, sleeping, and exercise habits along with adopting novel stress-relieving activities are more important now than they have ever been. As Stanford Medicine’s chief wellness officer Tait Shanafelt, MD, stated, “We should not be recycling the wellness offerings of the past, as if retooled versions of those approaches are the current needs…we need to approach this situation with fresh eyes, ask our people what they need, develop our response based on the needs they’ve expressed, and effectively and compassionately communicate with them.”^18^ Ensuring adequate and appropriate mental health care when needed may help physicians and HCWs develop improved emotional and cognitive resilience to withstand the impact of such traumatic events.

As cardiothoracic surgeons we are the natural leaders of our respective teams and the example-setters for others. Many of our staff are working from home, battling the challenges of how to home-school children or provide childcare while also working and without their normal work–home boundaries or work support system. Others face furlough, with the associated financial strain, along with physical isolation. Sincere gratitude from leaders and between coworkers can be a powerful source of support. Listening to our colleagues and specifically asking them about their concerns and needs are important steps. Although we are all physically distant, being intellectually and socially connected is essential for overall resilience and fortitude. Weekly leadership town halls, conference calls, large interactive webinars, and/or smaller scale virtual meetings allow our teams to stay informed and enable them to relay their evolving needs and concerns, and us to relay ours. The incorporation of non-work-related “joyful” experiences to balance professional and personal life is also desired and helpful. As Abigail Adams wrote to her son John Quincy Adams, “It is not in the still calm of life, or the repose of a pacific station, that great characters are formed,” rather “the habits of a vigorous mind are formed in contending with difficulties. Great necessities call out great virtues.”^19^ The importance of our leadership, compassion, and support in this turbulent time should not be underestimated.^17-19^

Shanafelt T, Ripp J, Trockel M. Understanding and addressing sources of anxiety among healthcare professionals during COVID-19 pandemic. *JAMA.* Published online April 7, 2020. PMID 32259193.

Goodwin, DK. Leadership. *Turbulent Times*. Simon & Schuster; 2018.

Choi KR, Heilemann MV, Fauer A, Mead M. A Second pandemic: mental health spillover from the novel coronavirus (COVID-19). *J Am Psychiatr Nurses Assoc.* 2020:1078390320919803.

11. ***What are the differences between pediatric and adult patients infected with COVID-19?***

While COVID-19 is a highly transmissible disease that infects most populations exposed, there exists significant variability in how the virus clinically manifests within a specific host. The age of the affected individual is now recognized as a significant factor in clinical presentation and impact. Infected children with COVID-19 do not have the same virulent respiratory syndrome as commonly seen in adults, although respiratory symptoms, along with fever and sore throat, are the most common presentations. Instead, they seem to have a less frequent (even rare), severe systemic inflammatory response that can occur weeks following initial exposure. Children can present with a rash on their hands and feet, diarrhea, vomiting, and hypotension. Multiple groups have developed evaluation protocols and classification schemes for assessing disease severity in the pediatric population. Unlike adults, the mortality in the pediatric population is extremely low and the majority of patients hospitalized have significant comorbidities. One theory that has been postulated, but not proven, is that the immaturity of the child’s immune system allows active resistance to the new organism, since all exposure is new. Adults do not have this innate, immature immune ability. Several other theories have been advanced, but only increased exposure and future trials will help us to better understand the differences between these two populations. Clinicians should also be aware of an increased incidence of a Kawasaki-like disease post COVID exposure that has been reported in children; the clinical significance of this remains to be defined.^7,20-22^

Ludvigsson JF. Systematic review of COVID-19 in children shows milder cases and a better prognosis than adults. *Acta Paediatr.* 2020;109(6):1088-1095.

Shekerdemian LS, Mahmood NR, Wolfe KK, et al. Characteristics and outcomes of children with coronavirus disease 2019 (COVID-19) infection admitted to US and Canadian pediatric intensive care units. *JAMA Pediatr.* 2020.

Hasan A, Mehmood N, Fergie J. Coronavirus disease (COVID-19) and pediatric patients: a review of epidemiology, symptomatology, laboratory and imaging results to guide the development of a management algorithm. *Cureus.* 2020;12(3):e7485.

Buonsenso D, Parri N, De Rose C, Valentini P. Toward a clinically based classification of disease severity for paediatric COVID-19. *Lancet.* 2020. Published online May 15, 2020.

12. ***How have some countries’ health care systems recovered from the COVID-19 pandemic: Experience from the other side of the “curve”***

Several countries, such as China and other Asian countries, have now experienced both sides of the “curve,” returning their health care systems to full capacity. The United States and other countries where the “peak” in the curve was more recent should learn from the successes and mistakes these countries have experienced. Many have publicly shared and published their observations and strategies to get back online. In a recent submission to *Lancet*, a consortium of 13 major heart centers in China analyzed their experience with increasing care. When these centers first started to increase care, testing for COVID-19 was insufficient; therefore, all 13 centers required 14-day quarantine. This made providing care to those in need cumbersome, with an increased risk of exposure and reported cases. Since that initial experience, testing programs became universal, with mandatory COVID-19 testing of all individuals who presented to the health care system, and the 14-day quarantine period was abandoned. A rapid return in both outpatient (clinic) and inpatient (procedural) capacity was reported in all centers. China’s numbers of COVID-19 cases in these facilities have remained near zero. The experience of these centers reinforces the key tenet of testing in the reactivation of services.^23^

Shi G, Huang J, Xiao D, Huang G, St. Louis J. Impact of COVID-19 outbreak on the congenital heart surgery program and the children after congenital heart surgery; an observations study from China. *Lancet.* 2020. IN PRESS.

## Summary

While there is no doubt that the post-COVID world, including that of the congenital heart surgery specialty, will never be the same, COVID presents a tremendous opportunity to emerge from this pandemic stronger, more resilient, more cohesive, and poised to provide superior care for our patients. As we learn how to effectively utilize telemedicine, we can effectively and compassionately care for them in a more convenient fashion. We are now more adept at evaluating and prioritizing the problems of our patients. As a result of this pandemic, the cardiothoracic surgery and cardiology/medical communities have come even closer together, many of our professional organizations have strengthened connections, and the advocacy arm of our governing organizations to support the needs of the medical community shined during our darkest hours with COVID. We have applied and can emerge with improved online medical education that touches the broadest audience—physicians, advanced practice providers, nurses, and HCWs all around the world.

Perhaps most importantly, COVID has tested our physical and emotional resilience and fortitude. The external world has seen and experienced the human side of the medical community and cardiothoracic surgery specifically. Our specialty has been most impressive. Congenital heart surgeons carry a broad set of skill sets and have been deployed to other areas of the hospital to provide help during the dearth of heart surgery. Cardiothoracic surgery is on the stage each and every day and the harder the act, the better our performance. The grit, professionalism, and empathetic side of the cardiothoracic surgeon have been projected to the outside world in a way that instills confidence, hope, and unity. It is a reminder to us why we went into this gratifying specialty in the first place.
